# The importance of correct implants positioning and masticatory load direction on a fixed prosthesis

**DOI:** 10.4317/jced.54489

**Published:** 2018-01-01

**Authors:** João-Paulo-Mendes Tribst, Vinicius-Aneas Rodrigues, Amanda-Maria-de Oliveira Dal Piva, Alexandre- Luiz-Souto Borges, Renato-Sussumu Nishioka

**Affiliations:** 1 DDs, MSc, PhD Student in Prosthodontics, Department of Dental Materials and Proshodontics, São Paulo State University (Unesp), Institute of Science and Technology, São José dos Campos / SP, Brazil. Address: Av Engenheiro Francisco José Longo, 777, Jardim São Dimas, São José dos Campos, São Paulo, Brazil; 2 DDs, MSc, PhD, Department of Dental Materials and Proshodontics, São Paulo State University (Unesp), Institute of Science and Technology, São José dos Campos / SP, Brazil. Address: Av Engenheiro Francisco José Longo, 777, Jardim São Dimas, São José dos Campos, São Paulo, Brazil; 3 DDs, MSc, PhD, Adjunct Professor, Department of Dental Materials and Proshodontics, São Paulo State University (Unesp), Institute of Science and Technology, São José dos Campos / SP, Brazil. Address: Av Engenheiro Francisco José Longo, 777, Jardim São Dimas, São José dos Campos, São Paulo, Brazil; 4 DDs, MSc, PhD, Adjunct Professor, Department of Dental Materials and Proshodontics, São Paulo State University (Unesp), Institute of Science and Technology, São José dos Campos / SP, Brazil. Address: Av Engenheiro Francisco José Longo, 777, Jardim São Dimas, São José dos Campos, São Paulo, Brazil

## Abstract

**Background:**

Through the biomechanical study of dental implants, it is possible to understand the dissipation effects of masticatory loads in different situations and prevent the longevity of osseointegration. Aims: To evaluate the microstrains generated around external hexagon implants, using axial and non-axial loads in a fixed four-element prosthesis with straight implants and implants inclined at 17°.

**Material and Methods:**

Three implants were modeled using CAD software following the manufacturer’s measurements. Then, implants were duplicated and divided into two groups: one with straight implants and respective abutments, and the other with angled implants at 17° and respective abutments. Both groups were arranged inside a block simulating bone tissue. A simplified fixed prosthesis was installed on both groups and the geometries were exported to CAE software. Five loads of 300N were performed at axial and non-axial points on the fixed prosthesis. Stress on the implants and strain on the block were both analyzed. An in vitro experiment was performed following all structures made in FEA in order to validate the model. In each experimental block, 4 strain gauges were linearly placed between the implants and the same loads were repeated with a loading applicator device.

**Results:**

The deformations computed by the gauges were correlated with the FEA results, showing that the group with inclined implants had more damaging biomechanical behavior and was significantly different from the group with straight implants (*P*<0.005).

**Conclusions:**

The mathematical model used is valid and inclined implants can induce unwanted bone remodeling.

** Key words:**Finite Element Analysis, Dental Implants, Fixed Prosthesis.

## Introduction

Based on the fundamental studies of Branemark *et al.* (1969) (1), and following a safe protocol of concepts, implant dentistry has established itself in modern dentistry as a tool of oral rehabilitation with reliable and safe results. In studying the longevity of the rehabilitative treatment, biomechanics has great importance in preventing already osseointegrated implant failure, since occlusal overload is one of the main causes of bone insertion loss around implants (2).

Bone structures have predictable behavior in front of a stimulus, as it has been defined that a normal mechanical stimulus results in preservation of bone tissue. Values considered low can lead to reabsorption due to disuse, and exacerbated values can lead to remodeling disorganization, which causes irreversible microstrain on the structure (3).

Several authors have studied the effect of the lever arm on implant prostheses and how this can influence the generated stresses (4,5). Finite element analysis (FEA) was defined as a useful system to predict the behavior of these stresses (4,5). However, a situation with inclined implants associated to a prosthetic lever has not been deeply studied in the literature yet.

In stress evaluation studies, the use of a bone tissue simulant material with the same mechanical behavior and capable of guaranteeing the system a reproducible pattern in all the specimens makes studies more concrete in providing inferences about the influence of the variables studied. In this context, polyurethane (6-10) is the material of choice due to its elasticity modulus and scientific validation. Thus, correlating two numerical methodologies to study stress allows for a concise direction to interpret the results for possible elucidation of the clinical occurrence.

Therefore, the use of Strain Gauge as a complementary method to FEA can improve the interpretation of the results (11,12).

Finally, the objective of this study was to evaluate the microstrains generated around fixed four-element prosthesis with straight implants and implants inclined at 17°, under axial and non-axial loads, and to verify if they are at the physiological limit.

## Material and Methods

-Tridimensional model

**Figure 1:**
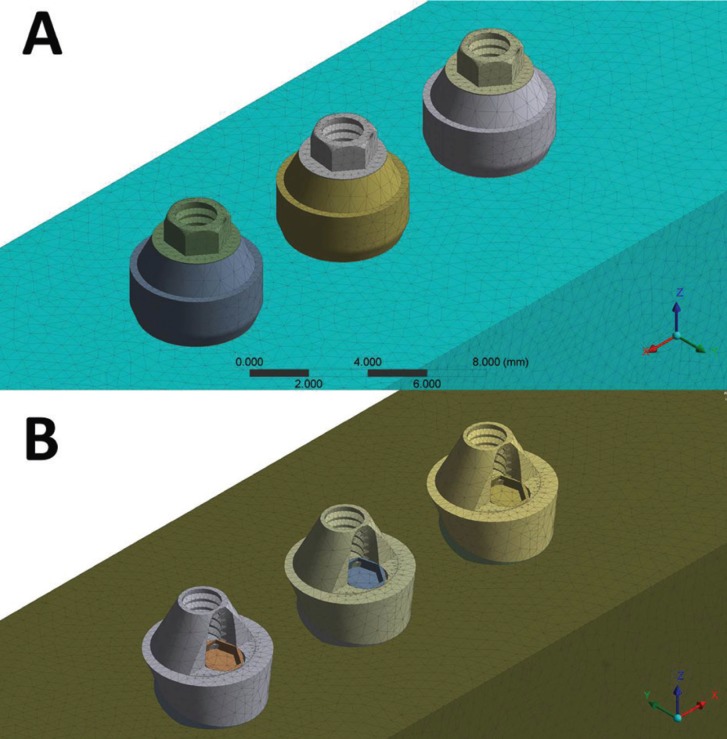
Final geometries according to the group: A) straight; B) inclined at 17 °.

Using Rhinoceros software (version 5.0 SR8, McNeel North America, Seattle, WA, USA), an external hexagon implant (3.75 x 13 mm) (AS TECHNOLOGY TITANIUM FIX - São José dos Campos, Brazil) was modeled. The external hexagon platform was 0.7 mm high and 4.1 mm in diameter. Next, the model was replicated

in order to obtain three identical implants with 3 mm distance between them. In the first group, the three implants were inserted without inclination, whereas the implants received a rotation of 17° for the second group (Fig. 1).

**Figure 2:**
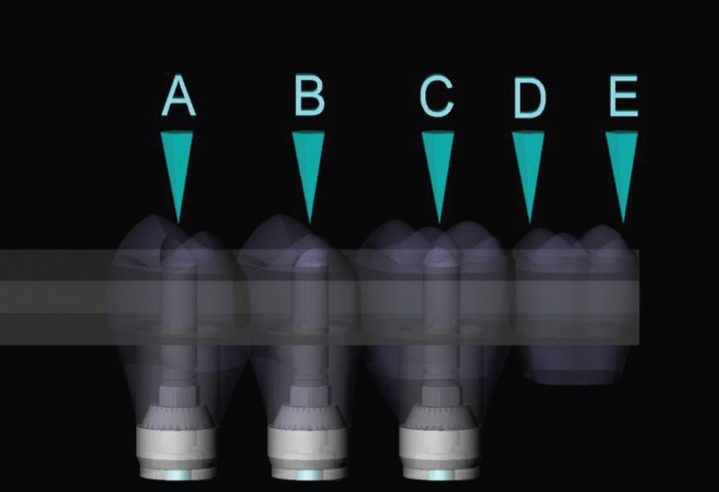
Fixed Prosthesis and load areas.

After dividing the groups, a mini conical abutment was placed on each implant. For the group with straight implants, the abutments presented centralized insertion and 3 mm band. For the inclined group, the abutments were tapered at 17° (allowing for correction of the insertion trajectory during prosthesis installation) and 2.5 mm band. An identical prosthesis for both groups was placed on the abutments (3 mm thick x 35 mm long x 15 mm wide). On the external surface of the fixed prosthesis, 5 circles of 2 mm diameter were demarcated to receive the load, corresponding to the center of the three retention screws (points A, B, C), 5 mm cantilever to point D, and 7 mm cantilever to point E (Fig. 2).

-FEA processing

The dimensions were imported into Ansys software (ANSYS 16.0, ANSYS Inc., Houston, TX, USA). The material properties were assigned to each solid component as isotropic, homogeneous and linearly elastic. Young’s modulus and Poisson’s ratio of the materials were reported ( Table 1) (13-15) and all contacts were considered bonded.

**Table 1:**
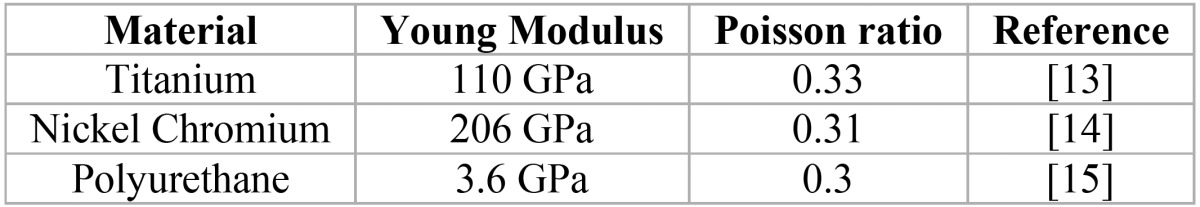
Properties of the materials used in the study.

-Mesh generation

A 10% convergence test determined 754,936 nodes with 440,893 elements for the straight group and 732,375 nodes with 428,219 elements for inclined group.

-Loading and fixations

The loading (300N on Z axis) was performed in the upper region of the fixed prosthesis, in 5 different regions located in supper surface from the prosthesis (Fig. 2). The location of the fixation was under the polyurethane block surface In all configurations, simulating the support of the sample on a plane.

-Experimental model

The model followed the same dimensions for all components of the system, based on the theoretical model of regular geometries. For simulation of the bone tissue, two blocks (95 x 45 x 30 mm) of polyurethane (F160 Axson, Cercy - France) were obtained through a rectangular stainless steel metal matrix. After polymerization of the polyurethane, the blocks were removed from the matrix and had their surfaces polished with sandpapers # 220 - # 600 under water.

For implant installation in the blocks, a set of milling cutters was used according to the manufacturer’s recommendations (AS TECHNOLOGY TITANIUM FIX

- São José dos Campos, Brazil). The matrices placed on the surface of the polyurethane following the methodology already used in other studies (7,16), serving as guides so that the implants were axially arranged and inclined at 17°. Three self-tapping implants of external hexagon measuring 3.75 in diameter by 13 mm in length (AS TECHNOLOGY TITANIUM FIX - São José dos Campos, Brazil) were installed in each block. Prosthetic abutments were installed on each implant with a torque of 20 Ncm with the aid of a manual torque wrench.

After placement of the implants and abutments, twenty fixed prostheses were cast in NiCr (n = 10).

-Strain Gauge (SG) installation

**Figure 3:**
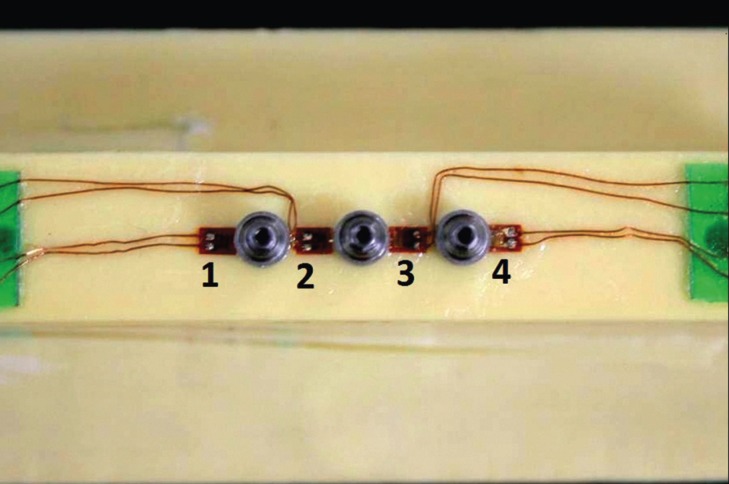
Strain Gauges linearly arranged between the abutments.

After careful cleaning of the blocks surfaces with isopropyl alcohol, four linear SGs (KFG-1-120-C1- 11L1M2R; KYOWA electronic instruments CO., Ltd., Tokyo, Japan, resistance 119.6 ± 0.4% Ω; gauge length: 1 mm; gauge factor: 2.08 ± 1.0%) were attached to each block with cyanoacrylate based adhesive. Two SGs were bonded to the proximal regions of the central implant and another two in the extremities, as shown in Figure 3. Evaluation of the resistance of each SG was performed through a multimeter device (Minida ET 2055: Minida São Paulo - Brazil). Bonding of terminal plates was made in the upper surface of the block, where the electrical connections were adapted. Variations of electrical resistance were converted into microstrain-rate units through an electrical signal conditioning apparatus (Model 5100B Scanner - System 5000 - Instruments Division Measurements Group, Inc. Raleigh, North Carolina, USA, FAPESP proc: 07 / 53293-4). Electrical cables allowed the connection between the SGs and the data acquisition apparatus, where the acquisition channels were installed.

-*In vitro* load application

Three static vertical loads of 300 N were performed (7,16) for each prosthesis for 10 seconds on all 5 points on the prosthesis surface similar to what was done in the previous computational study (Fig. 2).

-Statistical analysis

All SGs data were submitted to one-way ANOVA followed by Tukey test, with a significance level of 5% (Minitab software, version 17.3.0, 2016).

## Results

The results obtained from FEA followed maximum principal stress criteria for the non-ductile solids. As titanium is a friable material that fails by traction, the generated microstrains were evaluated for the block model.

**Figure 4:**
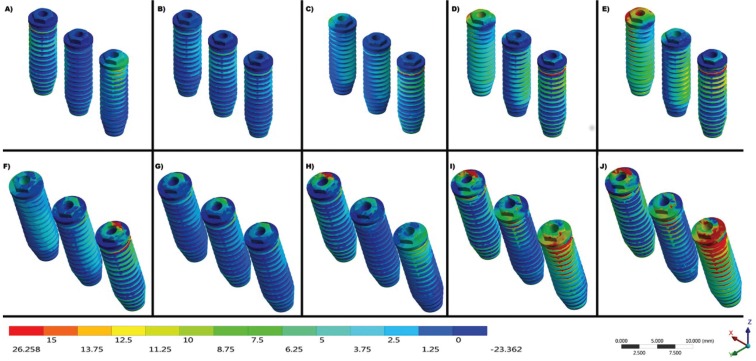
Maximum Principal Stress on implants under load aplications at points A, B, C, D and E, respectively. A-E) Straight Group and F-J) Inclined 17° Group.

The maximum stress found in the titanium implants during the different loads was expressed by the color scale in Figure 4.

**Figure 5:**
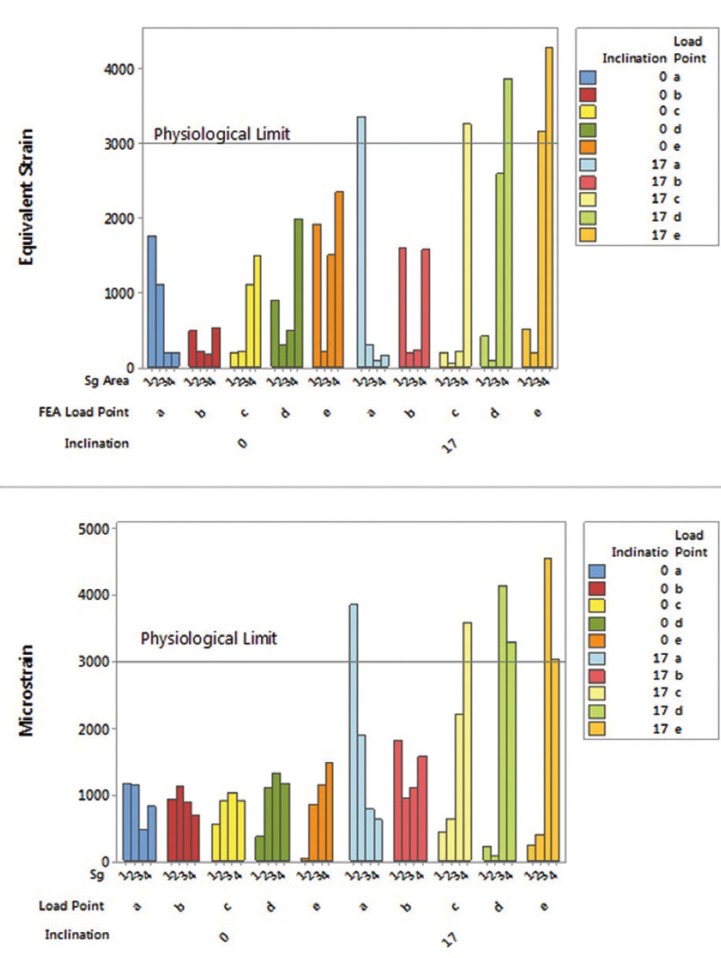
Bar graph of the strain generated in the in silico and *in vitro* experiment for both groups.

The values around the implants exhibit a behavior pattern that is accentuated the farther the force is applied in relation to the central implant. Also, the system angulation aggravated the generated strains. In the comparison between all the points of load application, the strain values around the implants were observed and are expressed in Figure 5. The group inclined at 17° presented higher values of stress around the implants and the bone, especially when the load application was nonaxial (Points D and E).

Data found on the blocks’ surface were analyzed by four different points of measurement for SG analysis of twenty samples. According to one-away ANOVA, the “implants inclination” factor was significant (*p* = 0.015).

One graph with the mean values for SGs according to the bone’s physiological limit was created (Fig. 5, microstrain). Another graph was created for correlating both methods and validating the mathematical model, thus presenting the equivalent strain by FEA (Fig. 5, equivalent strain).

In Figure 5, it is also possible to observe that the straight group and point B of the inclined group did not cross the physiological limit for both methods. Also, the graphs show that the stress peaks measured by FEA and SG are not identical; however, they exhibit the same mechanical behavior.

## Discussion

Stress distribution is an important factor that indicates the suitability of a fixed prosthesis and depends on the material properties and geometric configuration (17). The use of prosthesis with inclined implants can influence the rehabilitation treatment’s longevity.

The experiment results were statistically significant ( Table 2) regarding the influence of using straight or inclined implants, which corroborates with the results found by FEA when an increase in stress concentration is observed in the group with inclined implants on all load application points. As previously reported, there have been influences of different implant inclinations on the stress concentration through FEA (18-21) and SG (20,22,23).

**Table 2:**
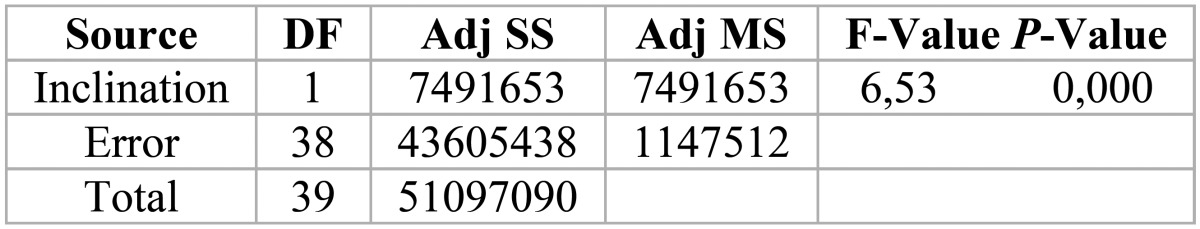
ANOVA - 1 way of the strain values (με) in the *in vitro* experiment (*p*<0.05).

Verifications on stress distribution in implants have been carried out using the FEA method in many studies (18,21). However, many of these models have not been validated and they can provide data that does not fit reality. FEA is an efficient methodology, economical and accurate tool (17) that allows for absolute values and stress distribution. However, due to the use of an ideal situation, an *in vitro* experiment is often necessary to confirm the results (9,11,12).

The main methods used to verify the stress generated in experimental models are Photoelasticity, Strain Gauge (SG) and Digital Image Correlation. From the methods mentioned above, SG is widely used in studies with implants and consecrated to obtain absolute values (9,12,17,22-25). SGs can be used in experimental trials to accurately measure surface parameters, but the measurement areas are strictly specific and unable to verify internal bone strain, which can be complemented by FEA (11,12). The association of these methodologies can prevent some disadvantages and can speed up the clinical time. Polyurethane has mechanical properties similar to human bone tissue ( Table 1), which allows for quantitative verification of the implants’ cervical region.

This is different from Elsyad *et al.* (2016) (23) who used SG in overdenture prostheses, but used acrylic resin as a substrate, only allowing for the qualitative results of inclined implants presenting higher stresses. The numerical stress analysis is not destructive, allows a correlation of two or more methodologies and allows clinical extrapolation with scientific bases (26).

Measurement of generated numerical strain makes it possible to predict the maintenance of alveolar bone with tensions between 1000 - 1500 με, and that loads above 3000 με will initiate a pathological reabsorption of the tissue (3,27-29). Within this established physiological limit, the graph from Figure 5 is marked with a line (Physiological Limit) to show the situations that make the system clinically unfeasible.

As with any laboratory study to maintain the principle of reproducibility scientific methodology, SG has limitations for the body in which the SGs are to be bonded.

Thus, the use of an isotropic material validated in the literature as a substitute for bone tissue in laboratory studies was successfully employed in several studies (9,12,20,26-29).

The main difference between the correlation obtained through the methodologies under study and the literature is the use of the same bone simulant material in both methods.

Using the same Young’s modulus narrows down the differences between them, so that the same biomechanical behavior can be verified for all specimens. Several authors have compared the material of the experimental study with a three-dimensional model containing cortical and medullar bone (9,12,20,26,28). In this way, they can be finding different values among methodologies that should find the same answer (20,26,28). Similar to this study simulated by FEA in which a resin was used to fix the implants in the in vitro analysis, Wu *et al.* (2016) (12) used a resin substrate to simulate human bone tissue in the SG analysis. Then, the authors also simulated the material in the analysis model using FEA, which guarantees better result precision.

Another way to guarantee significant results (*p*<0.05) was found by Eser *et al.* (2009) (11) that used SGs attached to cadaver jaws and then simulated this tissue in FEA. However, using a resinous material is less complicated and is easier to reproduce by other researchers.

The results found in the present study showed that the inclined implants in a fixed prosthesis promotes more microstrain than the physiological bone maintenance limit (Fig. 5). This is different from several authors that studied inclined unitary implants in the anterior region and did not find harmful strain values to bone tissue (18,20,30). This may be related to the fact that the anterior region does not have a masticatory load as high as the posterior region, which causes the inserted teeth and implants to receive less force. In addition, the force applied in this region is oblique along the axis of the implant and is better balanced with angled abutments arranged opposing it.

When we observe the implants’ platform in Figure 4, we can notice that the inclined group presents more traction and compression concentration with the same load in the straight implants. This can be explained by the fulcrum in the prosthesis being altered when the implants are inclined, suggesting that the force dissipation in non-axial components is more harmful.

The results of the present study at point C corroborate with previous research (9) that also used prosthesis on three implants and SG. They evidenced that the mesial of the last implant had higher strain values than the distal of the same. This behavior means that when a non-centered force is applied to the prosthesis, a fulcrum forms at the implant closest to the compression point, and increases as more distal points are used (D and E), generating a greater rotation tendency of the prosthesis and further damage to the surrounding tissue. However, even at the most distal point of the present study, if the implants were ideally placed in the tissue, the values of the physiological limit for unwanted resorption were not reached; unlike the group with implants inclined at 17° where the unique load point which did not represent major problems was axial point B, exactly at the center of the prosthesis.

Figure 5 shows the strain peaks occurred in the bone crest region for both methodologies, where the reabsorption and insertion loss of an already osseointegrated implant begins. The lowest strain value measured by both methodologies is when a fully axial force is applied at the center of the prosthesis, balancing the distribution throughout the system and maintaining the physiological limit. Similar to our study, several authors found quantitatively higher stress values in the peri-implant bone when subjected to non-axial loads were found (9,16).

The results found in points D and E for both groups resemble studies that analyzed the lever arm of implants and found higher stress values around the last implant (4). For the group with three inclined implants, it would not be indicated to perform a fixed prosthesis, since applying fully axial loads would be impossible in the oral medium.

As FEA presented strains close to in vitro measurements, the FEA model was considered validated, corresponding to the *in vitro* experiment. The difference between FEA and SG results may be due to SG measurement errors, as settings reported in the FEA model, as well as the scoring areas in the FEA and the experiment are not exactly identical.

Regarding limitations of this study, it can conclude that:

1. Strain and stress were significantly greater when inclined implants were used with any load application that is not fully centered;

2. The mathematical model used is valid for stress anal sis in implants and bone strains.
